# Photocatalytic Degradation of Bisphenol-A using N, Co Codoped TiO_2_ Catalyst under Solar Light

**DOI:** 10.1038/s41598-018-38358-w

**Published:** 2019-01-24

**Authors:** Alok Garg, Tejasvi Singhania, Ashutosh Singh, Shilpa Sharma, Sonam Rani, Ananya Neogy, Shri Ram Yadav, Vikas Kumar Sangal, Neha Garg

**Affiliations:** 1 Department of Chemical Engineering, Thapar Institute of Engineering & Technology, Patiala, 147004 India; 2 School of Chemistry and Biochemistry, Thapar Institute of Engineering & Technology, Patiala, 147004 India; 3 School of Basic Sciences, Indian Institute of Technology Mandi, Mandi, 175005 India; 4 School of Science, Sandip University, Nashik, Maharashtra 422213 India; 5 Department of Biotechnology, Indian Institute of Technology Roorkee, Roorkee, 247667 India; 6 Department of Chemical Engineering, Malaviya National Institute of Technology, Jaipur, India

## Abstract

Advanced oxidation processes (AOPs) including heterogeneous photocatalysis has proven as one of the best technique for waste-water treatment. Photocatalytic process using semiconductor like TiO_2_ based heterogeneous photocatalysis is a promising method for the treatment of toxic pollutants. In the present study, visible-light photoactive cobalt and nitrogen co-doped TiO_2_ nanoparticles were synthesized via wet impregnation method. The photocatalysts were characterized using X-ray diffraction (XRD), Raman Spectra, Fourier Transform Infrared (FTIR) Spectroscopy, Scanning Electron Microscopy (SEM), Transmission Electron Microscope (TEM), UV-vis spectrophotometer and X-ray photoelectron spectrophotometer (XPS). The photocatalytic activitiy of prepared (N, Co)-codoped TiO_2_ on the mineralization of Bisphenol-A (BPA) under visible light irradiation was studied and the results were compared to commercial TiO_2_ (Degussa P25). The results demonstrated that 1.5% Co and 0.5% N – codoped TiO_2_ samples revealed higher activity than commercial TiO_2_. Total organic carbon (TOC) removal was observed to be 97%, which indicate the complete mineralization of BPA. GC-MS analysis was carried to find out the possible intermediates formed and reaction pathway.

## Introduction

In today’s world poor sanitation, waterborne infections, water quality declination, and absence of clean water supply are a great concern arose due to increase in population. The impacts of chemicals like colors, herbicides, pesticides etc. discharge in streams and lakes that are suspected to be endocrine-disrupting chemicals (EDCs) are creating havoc on the biological systems. Despite of the fact that it is still perplex for analysts whether such chemicals have an impact upon people or not, nevertheless it is important to create productive strategies for degradation of these EDCs from wastewater^1^.

Bisphenol-A [2,2-bis (4-hydroxyphenyl) propane] or BPA is generally utilized as a beginning material for epoxy and polycarbonate plastics. BPA from plastic items into water has as of late been identified as a genuine reason of water contamination. Also, high concentrations of BPA can be contained in wastewater from its creation plants. BPA enters into the water bodies through generation units and by drainage made by BPA-based saps^2^. Microorganism can effectively degrade BPA but requires long time for the wastewater containing BPA. Therefore, we require the simple and cheap strategies for degradation of BPA in wastewater.

A number of physical, chemical, and biological techniques have been developed over the last two decades to remove toxicity from pharmaceutical wastewater but these treatment methods have also their disadvantages. These methods are not much efficient to bring down the pollution parameters to the satisfactory level. The current techniques for treatment relies on the development of receptive synthetic species, a method termed as advance oxidation processes (AOPs). AOPs are used for degradation of wastewater containing bio-recalcitrant organic pollutants or removal of pathogens. AOPs produced highly reactive chemical species like hydroxyl radicals that completely destroy the pollutants present in wastewater. The central point influencing the AOPs are pH, convergence of the waste to be dealt with, catalyst loading included, UV illumination and time^3^.

Titanium dioxide (TiO_2_) is one the most efficient phototocatalysts used for photocatalytic oxidation of organic pollutants present in wastewater^4^. Under UV irradiation, TiO_2_ is photo activated and active oxygen species such as hydroxyl radicals are formed on the surfaces of the TiO_2_ crystals. Most of the organic compounds could be decomposed into CO_2_ and H_2_O by the attack of these radicals that possess high oxidizing power. TiO_2_ photocatalysis in aqueous medium yields a variety of intermediates. It is chemically and photochemically stable, but is only excited by ultraviolet light having wavelength (λ) less than 390 nm, therefore the light utilization efficiency to solar irradiation and a fluorescent lamp is quite low. To conquer the portion of the troubles experienced, diverse dopants are being explored with the point of upgrading the morphology of TiO_2_ in the photocatalysis. Dopants adjust the electronic structure of TiO_2_ to widen its viable scope of light affectability for photocatalysis from the ultra-violet (UV) area to the distinctive light locale. Therefore, attempts were made to extend the absorption range of titanium dioxide into the visible-light region by the introduction of a donor level by transition metal doping. The main aim of these activities includes the (i) combination of energy levels into the band gap of TiO_2_, (ii) changing the life time of photogenerated charge carriers, (iii) swap of the Ti^4+^ with cation of the identical size, and (iv) shifting the VB and/or the CB in order to enable the process of photo-excitation at lesser energies, achievement of which depends on the method of preparation^5–7^. In order to alter the optical response of TiO_2_ photocatalysts, doping and codoping of titania is an effective method to alter the band gap energy. The main objective of doping is to decrease the band gap of TiO_2_, i.e., to induce a bathochromic shift and thus to extend its wavelength range response to the visible region^8^.

Various studies have been devoted to achieve desired band gap narrowing of titania by using non-metals such as nitrogen (N), phosphorous (P), sulfur (S), fluorine (F), and carbon (C)^9–15^. Nitrogen has been reported to be the most promising dopant as it can easily substitute oxygen (O) in the TiO_2_ lattice owing to its atomic size comparable with that of O, small ionization energy and high stability. Non-metal dopants P and S have also been reported to show optimistic outcomes for visible light activity in titania photocatalysts^4,16^. For the non-metal-doped titania photocatalysts, the mixing of N, S or C (2p) O (2p) states shifts the VB edge upwards, resulting in a decrease of the band gap of the N-doped TiO_2_ and thereby the photocatalyst can be energetic under visible light irradiation. The doping of a range of transition metal ions in to TiO_2_ could shift its optical absorption edge from UV to visible light range^17^. At a high dopant concentration, the metal ions can behave as recombination centers for the photoinduced charge carriers thereby, decreasing the quantum efficiency^16,18^. Vanadium^17,19^, copper^20,21^ and cobalt^22^ doping on TiO_2_ offers a possible promising strategy to enhance the characteristics of photocatalytic species and activity under visible light.

In an effort to study the effect of surface co-modifications on photocatalytic degradation and characteristic aspects of photo-induced charge properties and possible synergic effects between the introduced components, the proposed study was undertaken with following objectives: (i) to synthesize the Co, N codoped TiO_2_, (ii) characterization of synthesized codoped TiO_2_, and (iii) photocatalytic activity (Degradation of BPA) of Co, N codoped TiO_2_ under solar light.

## Results and Discussion

### Characterization of photocatalysts

#### X-ray diffraction (XRD)

**Figure 1:**
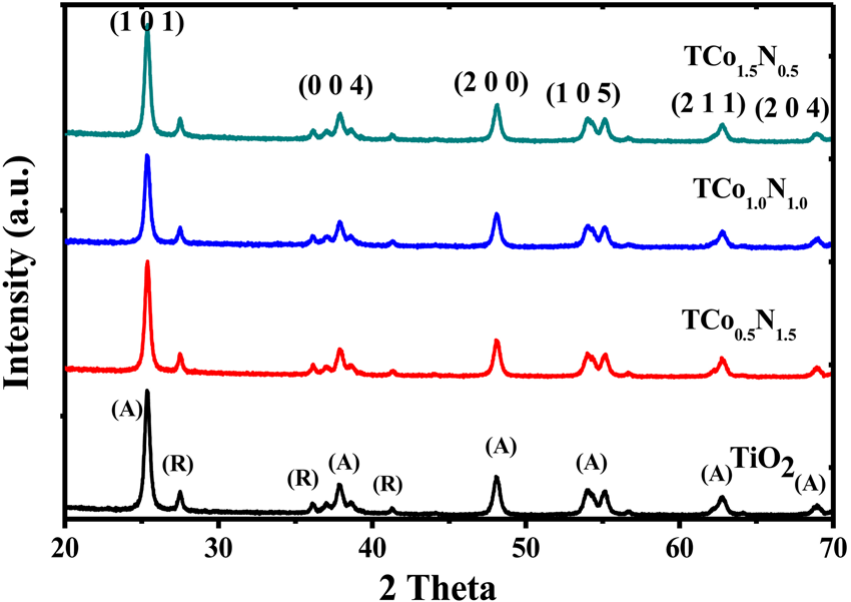
X-ray diffraction pattern of different photocatalysts.

The XRD patterns of N, Co codoped TiO_2_ catalyst indicates that the structure consists of anatase and rutile phases while undoped TiO_2_ exhibits the pure anatase phase as shown in Fig. 1. XRD peaks (1 0 1), (0 0 4), (2 0 0), (1 0 5), (2 1 1) and (2 0 4) were identified corresponding to anatase phase (ICDD No. 86–1048, 86–1157) and 2Theta = 27.4° corresponds to the rutile phase for codoped TiO_2_ photocatalyst^23^. The anatase and rutile phase contents of the codoped TiO_2_ were calculated by analyzing the intensities of anatase 101 peak at 2θ = 25.5° and rutile 110 peak at 2θ = 27.5°. The anatase % (A%) was found by the following equation:

1 $$Percentage\,of\,Anatase\,( \% A)=\frac{100}{1+1.265\frac{{I}_{R}}{{I}_{A}}}$$

2 $$Percentage\,of\,Rutile\,( \% R)=100- \% A$$

where I_A_ is the intensity of the 101 peak of anatase and I_R_ is the intensity of the 110 peak of rutile. From equations 2 and 3, anatase phase has been found to be 74.25% and rutile phase was 25.75% in the N, Co codoped TiO_2_ (Table 1). The average crystallite size of the sample has been estimated using Debye-Scherer equation:

3 $$d=\frac{0.89\lambda }{\beta \,\cos \,\theta }$$

where d represents the crystallite size, λ is the wave length of incident X-ray, β is the full width at half maximum, and θ represents the scattering angle. The mean grain size of the N, Co codoped TiO_2_ has been estimated as 3.5 nm by Debye-Scherer’s equation (Table 1).

**Table 1:** Physicochemical properties and calculated energy band gap of codoped TiO_2_ samples.

Catalyst sample	2 Theta (degree) (101)	d-spacing (Å)	Anatase lattice parameters (Å)		Phase		Energy band gap (eV)	Wavelength (nm)
			a = b	C	% A	% R		
T	25.377	3.5140	3.7822	9.5023	66.78	33.22	3.2	387
TCo_0.5_N_1.5_	25.325	3.5140	3.7822	9.5023	70.47	29.53	2.88	430
TCo_1.0_N_1.0_	25.308	3.5162	3.7842	9.5146	64.68	35.32	3.10	400
TCo_1.5_N_0.5_	25.325	3.5140	3.7822	9.5023	74.25	25.75	2.85	435

#### Raman Spectroscopy

**Figure 2:**
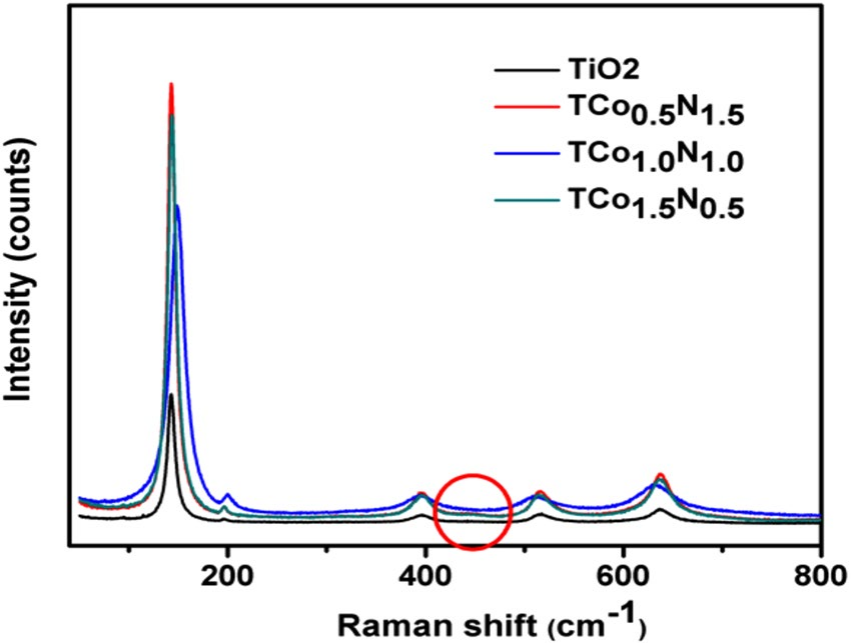
Raman spectra of TiO_2_ and its formulation.

Raman spectroscopy provides the important data for the presence of different phases in TiO_2_. The anatase phase of undoped. TiO_2_ has six Raman active modes in the vibrational spectrum centered around 144 cm^−1^, 197 cm^−1^, 399 cm^−1^, 513 cm^−1^, 519 cm^−1^, and 639 cm^−1^ corresponding to E_g_, E_g_, B_1g_, A_1g_, B_1g_ and E_g_ respectively^24^ whereas the rutile TiO_2_ shows four Raman-active fundamental modes at around 143 cm^−1^, 447 cm^−1^, 612 cm^−1^ and 826 cm^−1^ corresponding to B_1g_, E_g_, A_1g_, and B_2g_ respectively for first-order effect^25^. In the present study, the Raman spectra measured confirmed the anatase phase for undoped TiO_2_ and gives the bands at 143 cm^−1^ (E_g_), 197 cm^−1^ (E_g_), 399 cm^−1^ (B_1g_), 517 cm^−1^ (A_1g_ + B_1g_), and 639 cm^−1^ (E_g_) whereas the doped TiO_2_ gives similar bands as of undoped TiO_2_ with an additional small band at 447 cm^−1^ (E_g_) confirming the presence of anatase and rutile phase in the codoped TiO_2_ (Fig. 2). As seen from Fig. 2, the signature peak of TiO_2_ in the codoped sample is significantly shifted, compared to that of the commercial TiO_2_ sample, which may be ascribed to the reduction in crystallite sizes in the codoped TiO_2_^26^. The reduction in the size of the particles also been confirmed by the help of SEM data. The obtained results from the Raman spectra are in perfect agreement with the literature reports and also corroborate well with the powder XRD and TEM results.

#### Fourier transform infrared (FTIR) spectroscopy

**Figure 3:**
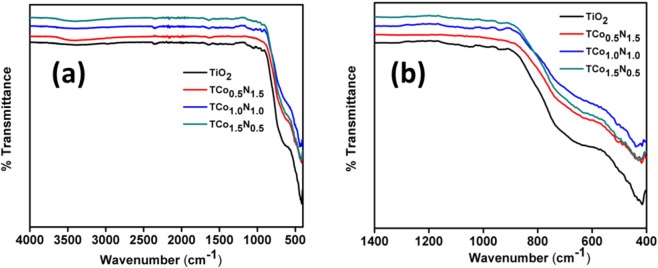
FTIR spectra (**a**) whole, and (**b**) figure print region of (**a**) TiO_2_, (**b**) TCo_0.5_N_1.5_, (**c**) TCo_1.0_N_1.0_ and (**d**) TCo_1.5_ N_0.5_.

FTIR patterns of the doped and codoped TiO_2_ particles were taken to get the information on the surface chemistry of the particles as shown in Fig. 3. The bands were observed in the range of 3670–3000 cm^−1^ and 1576–1710 cm^−1^ in undoped and all codoped TiO_2_ particles and were attributed to -OH stretching vibration and -OH bending respectively. Additionally, a band in the fingerprint region around 560–760 cm^−1^ in all the samples was attributed to the Ti-O-Ti stretching vibration^27^. FTIR pattern did not exhibit any band equivalent to the doped or co-doped metal oxide due to their low weight percentage.

#### Scanning Electron Microscopy (SEM)

**Figure 4:**
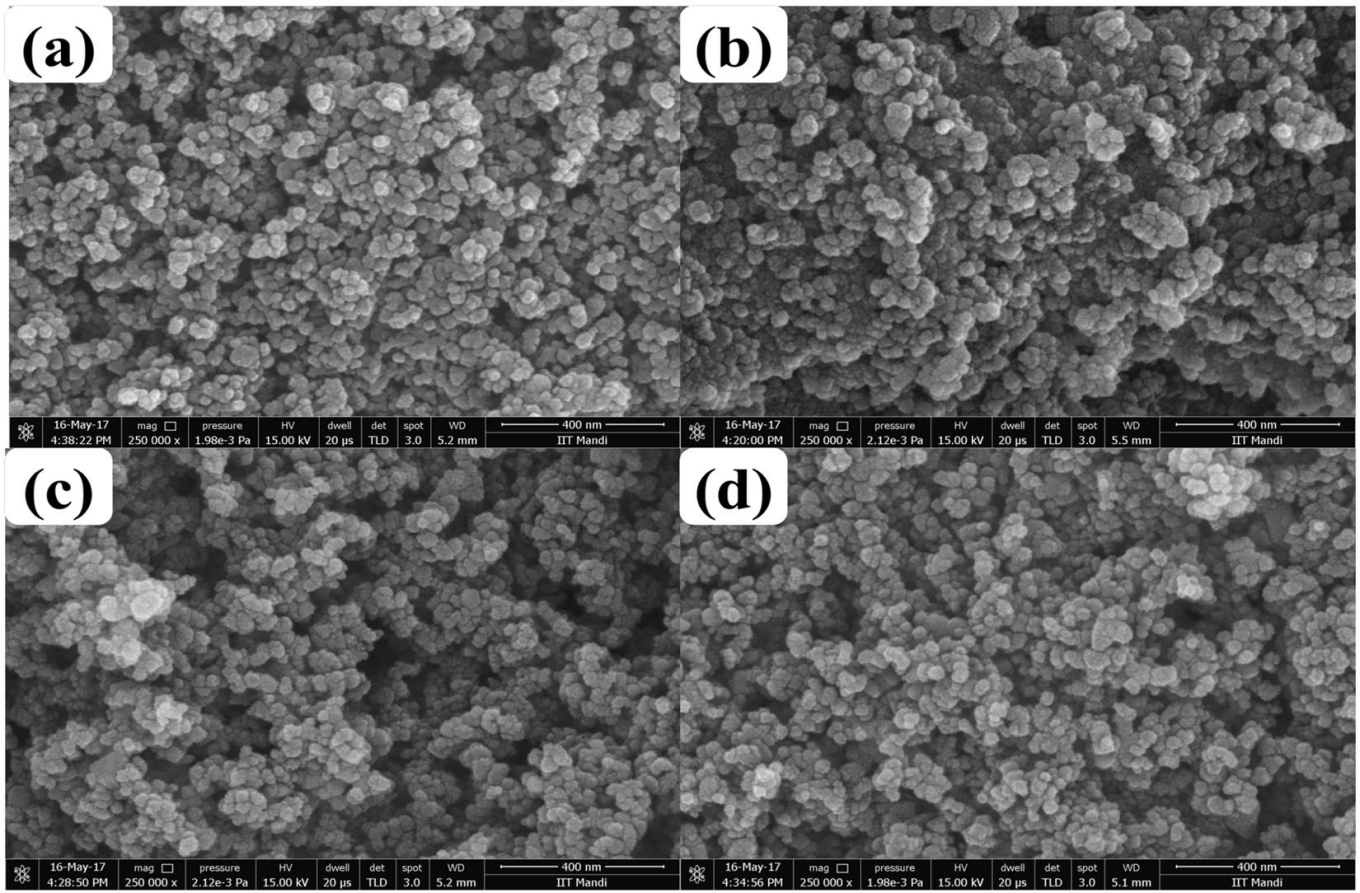
SEM images of (**a**) TiO_2_, (**b**) TCo_0.5_N_1.5_, (**c**) TCo_1.0_N_1.0_ and (**d**) TCo_1.5_ N_0.5_.

Figure 4 shows typical SEM micrographs of TiO_2_, and codoped TiO_2_. A detailed SEM investigation of the particle surfaces states that the primary particles are quite uniform in size and roughly spherical in shape, and that the agglomerates are fused together to form relatively larger uneven grains. Moreover, all samples display a narrow size distribution for the primary particles. All SEM results are in good agreement with the XRD data.

#### Transmission electron microscopy (TEM)

**Figure 5:**
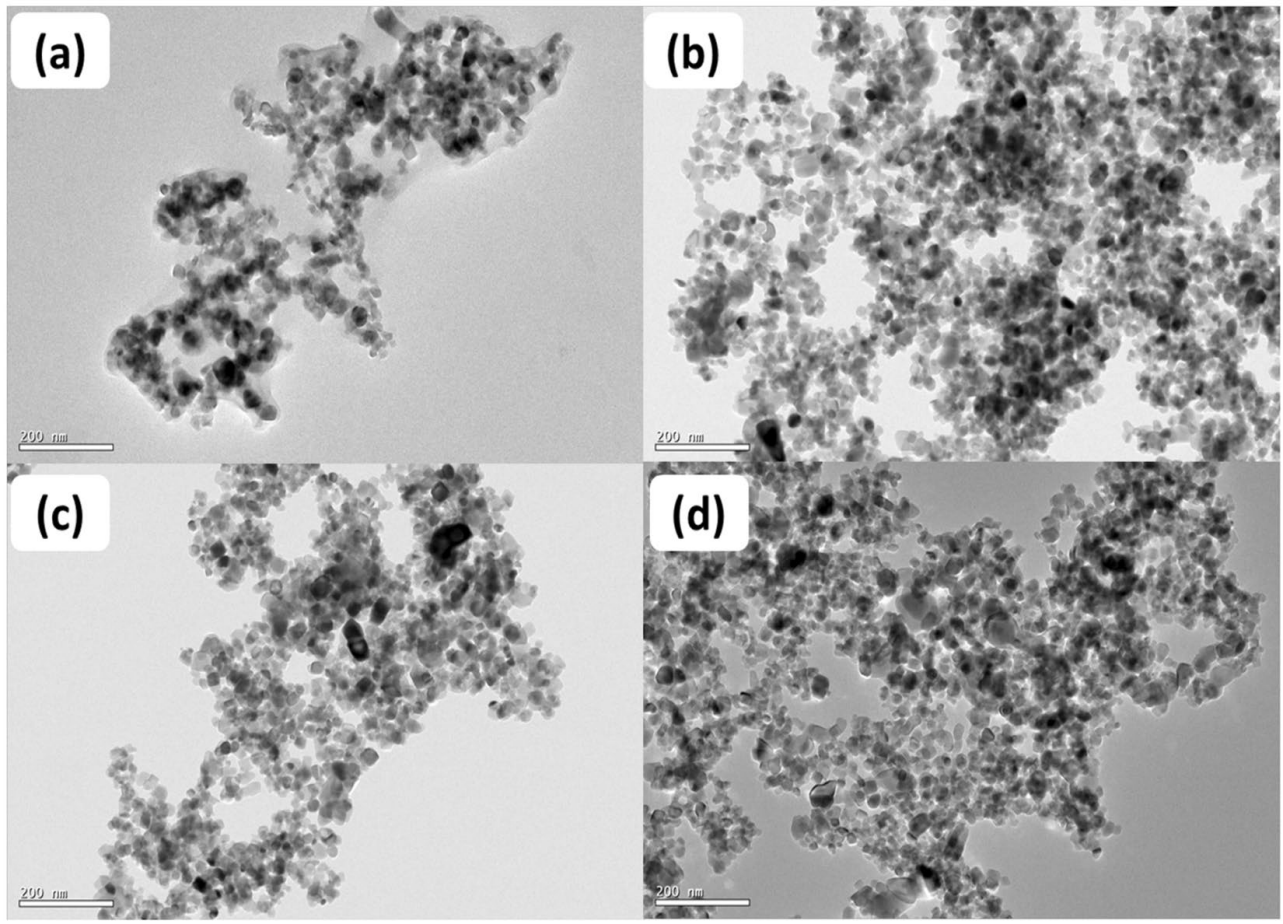
TEM images of (**a**) TiO_2_, (**b**) TCo_0.5_N_1.5_, (**c**) TCo_1.0_N_1.0_ and (**d**) TCo_1.5_ N_0.5_.

**Figure 6:**
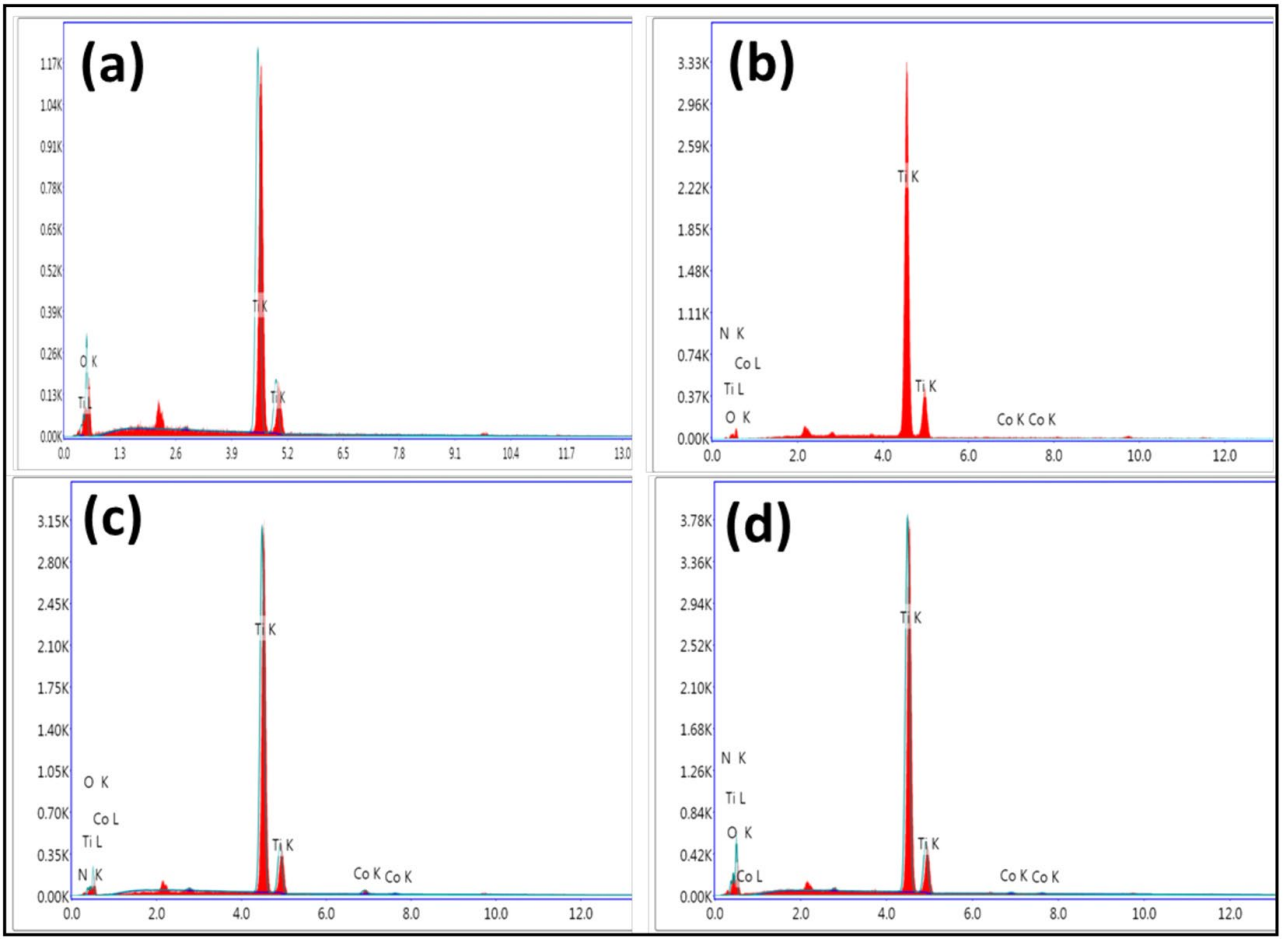
EDX mapping of (**a**) TiO_2_, (**b**) TCo_0.5_N_1.5_, (**c**) TCo_1.0_N_1.0_ and (**d**) TCo_1.5_ N_0.5_.

The TEM was used to observe the morphological and uniformity of structure of the particles. All the doped and codoped particles were small and nearly spherical in shape as shown in Fig. 5. It can be seen that the particle size estimated from the TEM data agrees well with the aforesaid XRD data. Further the incorporation of Co and N with TiO_2_ in the codoped particles was also confirmed by elemental analysis using energy dispersive X-ray analysis (EDX) (Fig. 6).

#### UV-Visible spectra

**Figure 7:**
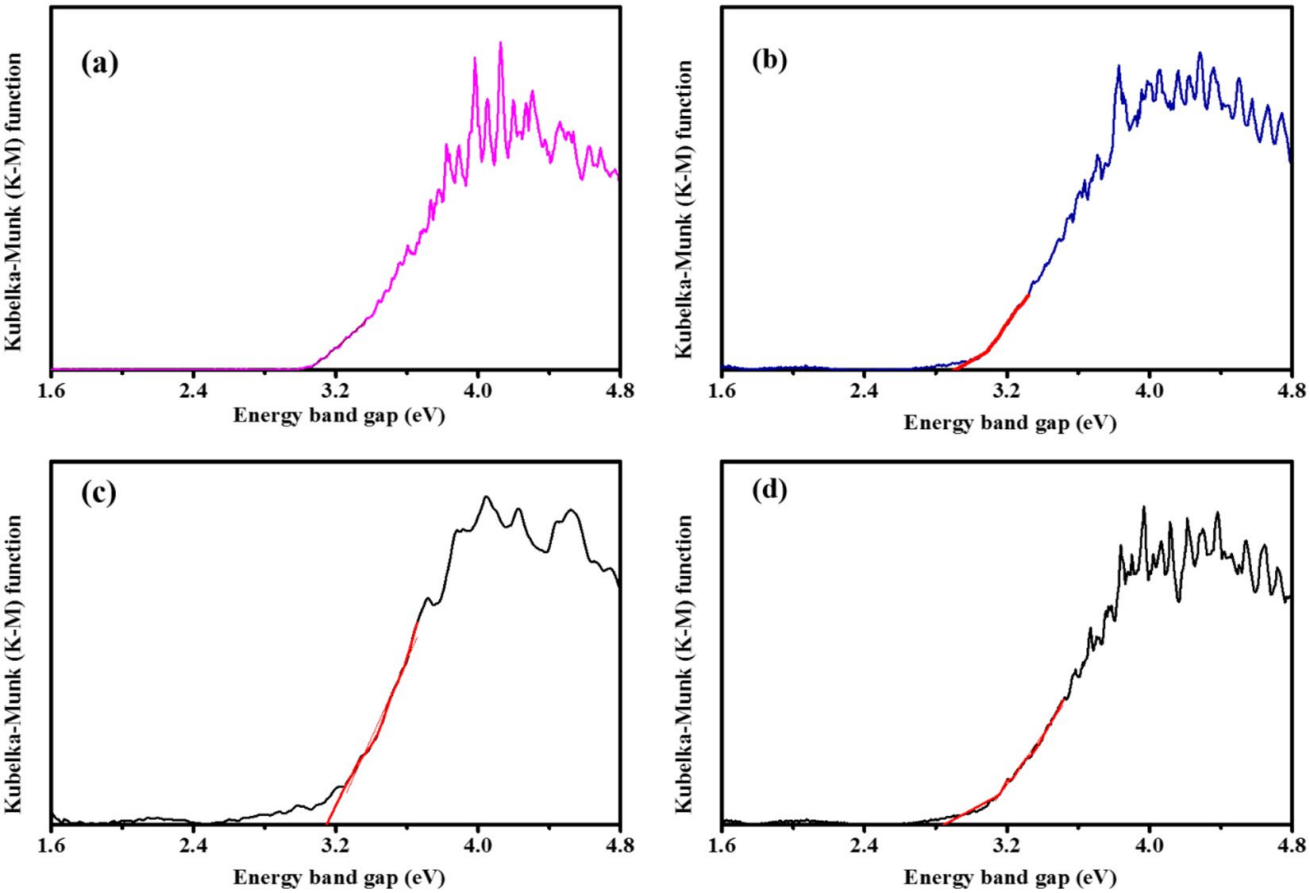
Kubelka-Munk (K-M) functionplot (**a**) TiO_2_, (**b**) TCo_0.5_N_1.5_, (**c**) TCo_1.0_N_1.0_ and (**d**) TCo_1.5_ N_0.5_.

Optical properties of Co and N codoped TiO_2_ were studied using UV–visible spectroscopy by measuring optical spectra in the range of 200–800 nm, in diffuse reflectance mode. Kubelka -Munk (K-M) plot (Fig. 7) was used to assess the band gap energies by extrapolating the linear region of the plot to intersect the photon energy axis; the obtained values are concise in Table 1. For pure TiO_2_, the band gap value of 3.2 eV was obtained, which is close to the expected value of the anatase phase (3.18 eV). After Co and N codoping, the band gap of TiO_2_ decreases. On the other hand, substantial narrowing of band gap was perceived after Co and N codoping.

#### X-ray Photoelectron Spectroscopy (XPS)

**Figure 8:**
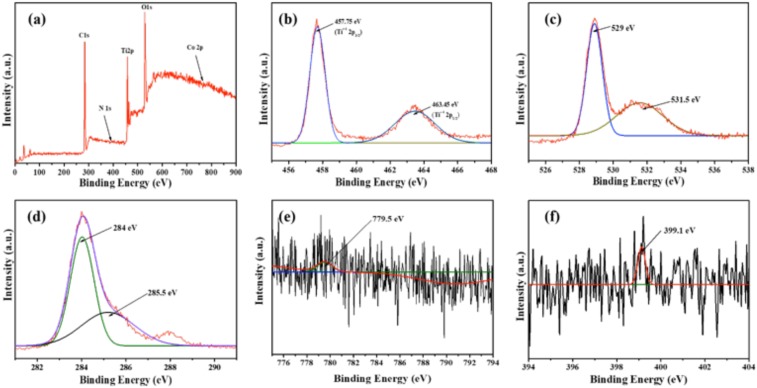
XPS of TCo_1.0_N_1.0_ sample (**a**) whole, (**b**) Ti 2p, (**c**) O 1s, (**d**) C 1s, (**e**) Co 2p3 and (**f**) N 1s.

X-ray photoelectron spectroscopic analysis was performed on the synthesized catalysts in order to confirm the presence of co-dopants and to decipher the detailed chemical state information of Co, O, N, and Ti and their oxidation states. Figure 8(a) gives the total survey spectrum which indicates the existence of Ti^4+^, O^2−^, N^3+^ and Co^2+^ in the TCo_1.0_N_1.0_ catalyst prepared. Figure 8(b–f) gives the XPS data for the elements Ti 2p, O 1s, C 1s, Co 2p3, and N1s. The XPS spectrum of other samples (pure TiO_2_, TCo_0.5_N_1.5_, TCo_1.0_N_1.0_, and TCo_1.5_N_0.5_) were shown in Figs S1–S3.

For the Ti 2 p region (Fig. 8(b)), the peaks of Ti sp_3/2_ and Ti 2p_1/2_ at 457.75 eV and 463.45 eV, respectively, they are all in good agreement with the values of Ti^4+^. The similar observations have been reported by Apiwong-ngarm *et al*.^28^ (i.e. the peaks of Ti sp_3/2_ and Ti 2p_1/2_ at 458.62 eV and 464.36 eV, respectively) and Wang *et al*.^29^ (i.e. the peaks of Ti sp_3/2_ and Ti 2p_1/2_ at 458.6 eV and 464.3 eV, respectively). No broad FWHM of Ti 2p_3/2_ peak signals also indicates the only presence of Ti^4+^ species^29^. The O 1s binding energies (Fig. 8(c)) of all the samples are located at 529 eV, which is assigned to bulk oxide (O^2−^) in the TiO_2_ lattice. Apiwong-ngarm *et al*.^28^ and Zhou *et al*.^30^ reported the similar peaks for O 1s. The signals of Co 2p3 (Figure (e)) were found to be weaker than all the others, due to the low doping amount. Co 2p_3/2_ peak located at band energies 779.5 eV was ascribed to the presence of Co_2_O_3_ or mixed valent Co2O3 with binding energy 779.4 eV reported in literature^31^. Figure 8(f) shows the XPS spectrum for N 1s. The peak at 399.1 eV demonstrated that nitrogen is incorporated into the TiO_2_ lattice. This considered to be the evidence of the presence of the Ti–N bond^32^.

### Photocatalytic activity

#### Effect of photolysis and adsorption

**Figure 9:**
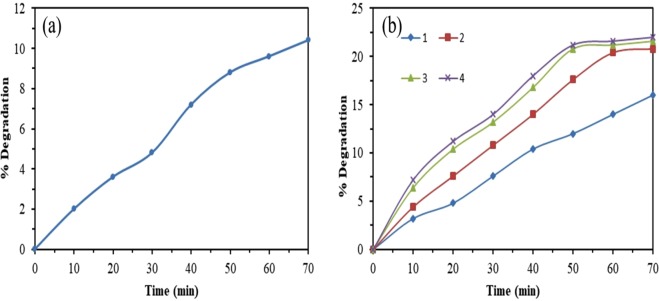
(**a**) Photolysis and (**b**) Adsorption of BPA with (1) TiO_2_, (2) TCo_0.5_N_1.5_, (3) TCo_1.0_N_1.0_ and (4) TCo_1.5_ N_0.5_.

To evaluate the effect of photolysis and adsorption on photocatalytic transformation, a series of preliminary experiments were carried out. Photolysis experiments were performed at pH = 5 and concentration of codoped TiO_2_ 70 mg *l*^−1^ with an initial concentration of BPA 50 mg *l*^−1^ for 70 min under solar light. As Fig. 9(a) shows, the photolysis of BPA was 11% only. The present results are in agreement with observation made by Rosenfeldt *et al*.^33^, which reported that photolysis of BPA during short time irradiation was insignificant.

On the other hand, adsorption capacity of the catalyst under dark conditions was observed at pH 5 and 50 mg *l*^−1^ initial concentration of BPA with 70 mg *l*^−1^ initial concentration of catalyst for 70 min reaction time. The percentage degradation due to adsorption of TiO_2_, TCo_0.5_N_1.5_, TCo_1.0_N_1.0_ and TCo_1.5_ N_0.5_ was 16, 20, 21 and 22% respectively (Fig. 9b).

#### Effect of pH

**Figure 10:**
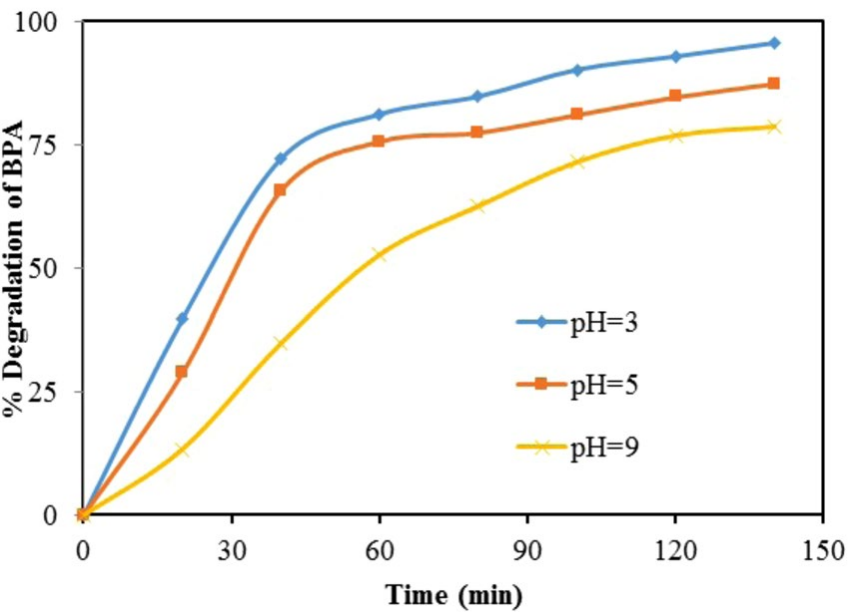
Effect of pH on initial rate of degradation of BPA: [TiO_2_]_0_ = 70 mg*l*^−1^, initial concentration of BPA = 20 mg *l*^−1^.

pH is an important factor in the degradation process. For estimating the optimum pH for the photocatalytic degradation of BPA using codoped TiO_2_, three different solutions (water containing BPA) have been prepared for which pH were maintained at 3, 5, and 9 respectively. Dose of doped TiO_2_ catalyst and initial concentration of BPA were 70 mg *l*^−1^ and 20 mg *l*^−1^ respectively. It was observed that the degree of disappearance of BPA is quite strong in acidic pH conditions (Fig. 10). The possible explanation of this BPA disappearance at pH = 3 is the amphoteric behavior of semiconducting material and the change of the surface charge properties of TiO_2_ photocatalyst.

#### Effect of catalyst dose

**Figure 11:**
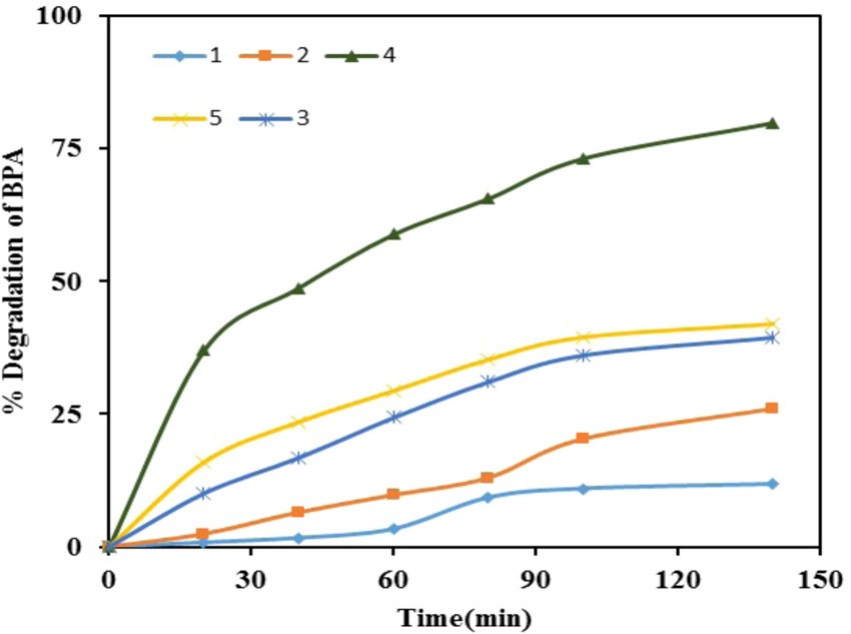
Effect of catalyst concentration on initial rate of degradation of BPA: pH = 3, initial concentration of BPA = 20 mg *l*^−1^, [TiO_2_]_0_ = (1) 35 mg *l*^−1^, (2) 70 mg *l*^−1^, (3) 105 mg *l*^−1^, (4) 140 mg *l*^−1^ and (5) 175 mg *l*^−1^.

For analyzing the effect of catalyst dose, experiments were performed with different doses of photocatalyst. In above experiments we used 70 mg *l*^−1^ TiO_2_ photocatalyst, but herein we have checked 35 mg *l*^−1^, 105 mg *l*^−1^, 140 mg *l*^−1^, and 175 mg *l*^−1^ TiO_2_ photocatalyst (Fig. 11) to find the effect of catalyst dose. It is clear from Fig. 11 that the maximum degradation of BPA was observed at the TiO_2_ concentration of 140 mg *l*^−1^. Moreover, on further increasing the photocatalyst dose until 175 mg *l*^−1^, the degradation of BPA decreases. It is due to the greater amount of catalyst, which causes increase in turbidity and thereby it impedes the penetration of light in the reactor, which in turn lowers the photo catalytic efficiency in the given working conditions. Another possible reason for the decrease in rate could be due to the decrease in the portion of the irradiated surface of the catalyst particle due to the obstruction of light in the dense slurry.

#### Effect of concentration of BPA

**Figure 12:**
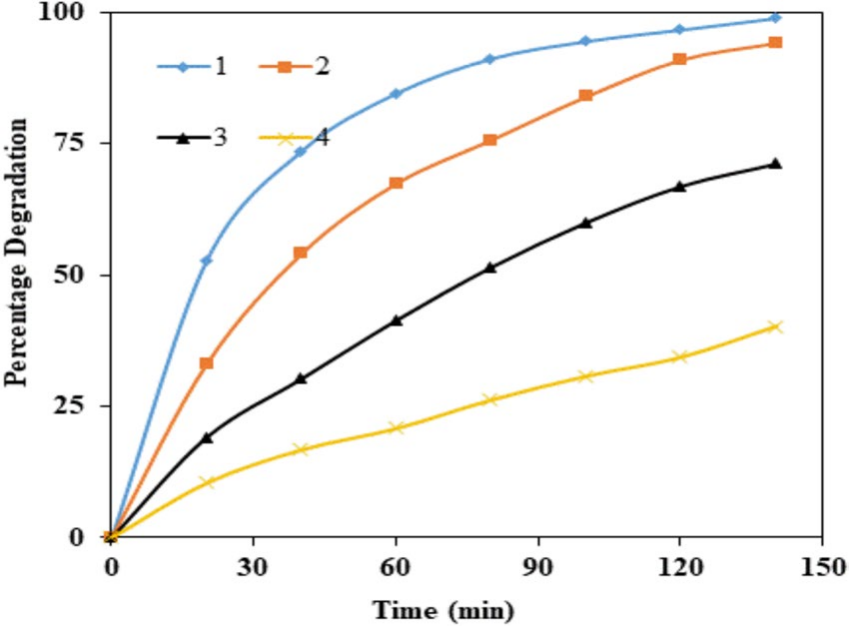
Effect of initial concentration of BPA for the degradation of BPA: pH = 3, catalyst concentration = 140 mg *l*^−1^, [BPA]_0_ = (1) 10 mg *l*^−1^, (2) 30 mg *l*^−1^, (3) 40 mg *l*^−1^, and (4) 50 mg *l*^−1^.

The effect of the initial concentration of BPA on the degradation of BPA under the solar light was determined. The obtained results have been presented in Fig. 12. The results indicated that the decomposition rate of BPA strongly depends on the initial BPA concentration. The efficiency of photodegradation of BPA decreased with increasing the initial BPA concentration. BPA with 10 mg *l*^−1^ shows 98% degradation after 140 min. On increasing the concentration of BPA until 50 mg *l*^−1^ the photodegradation became very slow, presenting a degradation of only 40%. As the initial concentration of BPA increased, more BPA molecules were adsorbed on the surface on the catalyst occupying the active sites and therefore the generation of hydroxyl radicals was reduced^34–36^. An increase of the initial BPA concentration results in an increase of the amount of BPA adsorbed on the catalyst surface, affecting the catalytic activity of the photocatalyst^37,38^. Moreover, the reduction of the light path length as the concentration increases could be one of the reasons for decreased catalytic acitivity.

#### Effect of dopant content

**Figure 13:**
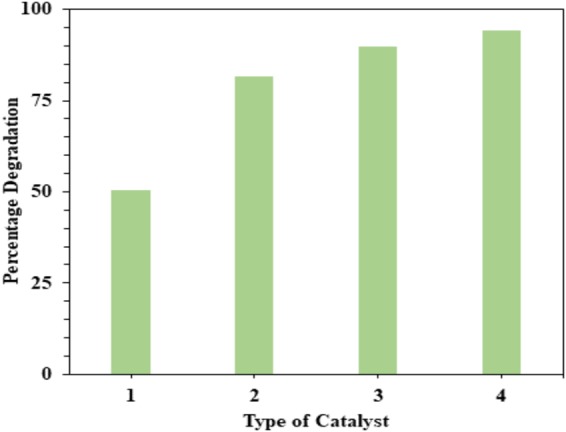
Effect of dopant content for the degradation of BPA: pH = 3, catalyst concentration = 140 mg *l*^−1^, and [BPA]_o_ = 30 mg *l*^−1^ (1) TiO_2_, (2) TCo_0.5_N_1.5_, (3) TCo_1.0_N_1.0_ and (4) TCo_1.5_ N_0.5_.

Cobalt and nitrogen codoped titania photocatalysts containing different amounts of codoping metal/nonmetal (TCo_x_N_y_, x = 0.5, 1 and 1.5 and y = 1.5, 1.0 and 0.5 respectively) were tested for the degradation of BPA under solar light UV irradiation. The percentage degradation of BPA vs. cobalt and nitrogen content were presented in Fig. 13. Under analogous conditions, photoactivity was low for pure TiO_2_ and 0.5% Co content, whereas with 1% of Co codopant in the material lead to an increase in BPA degradation. The photocatalytic degradation of BPA increases for higher (>1%) Co doping levels (Fig. 13). Regarding BPA degradation rates, a maximum degradation rate was observed for 1.5% Co codoping (i.e. TCo_1.5_ N_0.5_).

#### Reaction kinetic studies

A simple power law kinetic model could relate the degradation of BPA. The pseudo first order kinetics in terms of degradation of BPA can be written as:

$$\frac{-\,d[C]}{dt}=k^{\prime} [C]$$

where k′ is the pseudo first order rate constant.

Integration (with the limit of C = C_o_ at t = 0) with C_o_ being the equilibrium concentration of the bulk solution, ln (C_o_/C) = k′t, where, C_o_ is the equilibrium concentration of BPA and C is the concentration of BPA at time t.

**Figure 14:**
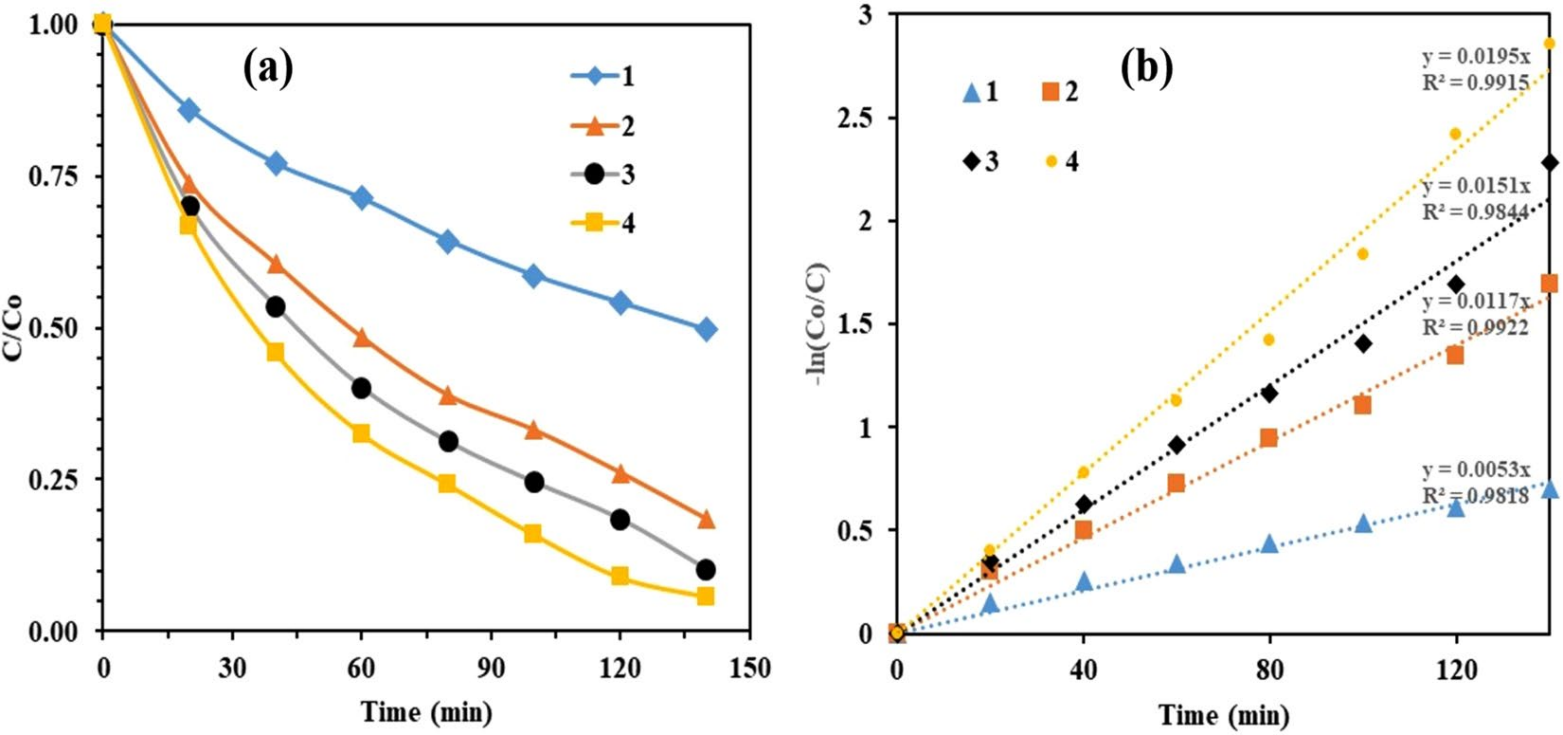
(**a**) Degradation and (**b**) reaction kinetics of BPA: pH = 3, catalyst concentration = 140 mg *l*^−1^, and [BPA]_o_ = 30 mg *l*^−1^ (1) TiO_2_, (2) TCo_0.5_N_1.5_, (3) TCo_1.0_N_1.0_ and (4) TCo_1.5_ N_0.5_.

A plot of C/C_o_ versus *t* for degradation of BPA has been shown in Fig. 14(a). A linear relationship was observed between degradation rate of BPA and irradiation time (Fig. 14(b)). The kinetic constants are 0.0053, 0.0117, 0.0151, and 0.0195 min^−1^ for TiO_2_, TCo_0.5_N_1.5_, TCo_1.0_N_1.0_, and TCo_1.5_ N_0.5_, respectively. Similar observation has been reported by Sharma *et al*.^2^ for the photo-oxidation of BPA with hydrogen peroxide (H_2_O_2_) and sodium persulfate.

**Figure 15:**
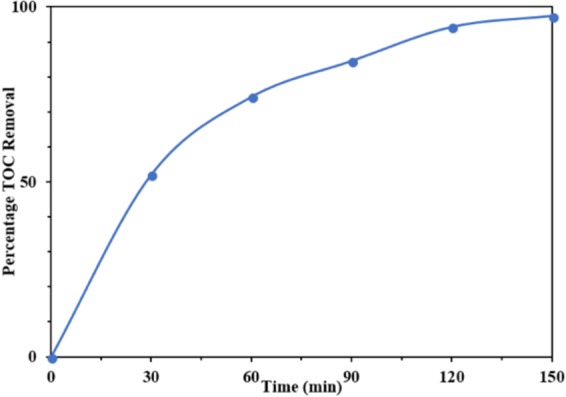
TOC removal versus time of BPA: pH = 3, catalyst (TCo_1.0_N_1.0_) concentration = 140 mg *l*^−1^, and [BPA]_o_ = 30 mg *l*^−1^.

In the direction of complete mineralization of BPA and the total degradation during photocatalysis, the total organic carbon (TOC) removal was estimated and has been shown in Fig. 15. The TOC removal showed a good mineralization trend of BPA. In the first 30 minutes of irradiation 52% TOC removal was observed and in the following 140 minutes, 97% TOC removal was observed. It indicates the complete mineralized of BPA at the end of 140 minutes.

#### Photodegradation pathway of BPA using codoped TiO_2_

**Figure 16:**
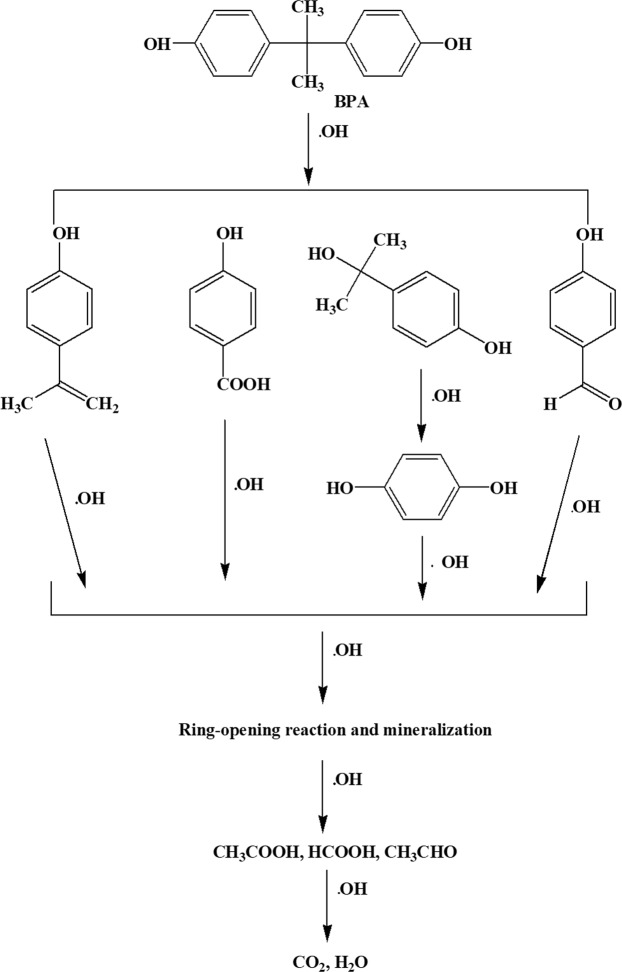
Proposed solar photocatalytic degradation reaction pathway of BPA.

The intermediate products formed during the photocatalytic degradation of BPA were identified from the Gas Chromatography – Mass Spectroscopy (GC-MS) analysis as shown in Table 2. Based on the intermediates found during GC-MS analysis, the proposed four possible reaction pathways has been shown in Fig. 16. It was noticed that other chromatographic peaks were also established but could not be positively recognized than the successfully detected compounds, (i.e. the match factor of the spectrum was not significant). It was clear that phenoxyl radicals was produced from all four of initial reactions. These single aromatic intermediates were presumably further oxidized through ring breaking reactions into non-toxic aliphatic acids. Finally, these aliphatic acids were oxidized to carbon dioxide and water.

**Table 2:** Intermediate compounds recognized from GC-MS analysis of photo-degradation of BPA.

Retention time (min) (90 min sample)	m/Z	Prominent intermediate compound formed
9.9	136	
15.698	140	
16.11	154	
17.36	136

## Conclusion

Co/N co-doped TiO_2_ with variable dopant composition have been synthesised by wet impregnation method. Catalysts exhibited changes in properties that could be associated with changes in structure. Dopants like cobalt and nitrogen were observed to disturb the physical properties of the nano particles, producing alterations in crystal structure, energy band gap as well as elemental composition. A decent photocatalytic rate was found by increasing the cobalt composition as a dopant for the degradation of BPA. TOC results revealed the complete mineralization of BPA. GC-MS analysis suggests the possible reaction pathway as well as the intermediates produced during the photocatalytic reaction.

## Materials and Methods

### Materials

Bisphenol-A [2,2-bis (4-hydroxyphenyl) propane or BPA] (C_15_H_16_O_2_) was procured from Sigma Aldrich. Titanium dioxide (Degussa P25) was used as catalyst and obtained from Evonik Degussa Corporation, USA. Cobalt (II) Nitrate Hexahydrate [Co (NO_3_)_2_.6H_2_O] and Urea were procured from Sigma Aldrich and were utilized as dopant. Hydrochloric acid (HCL) and sodium hydroxide (NaOH) were purchased from S. D. Fine Chemicals Limited, India and utilized to maintain the pH. For all the tests, distilled water (DW) and methanol were utilized as a solvent.

### Preparation and characterization of codoped TiO_2_

The codoped TiO_2_ nano powder was prepared using the method discussed by Garg *et al*.^23^ with a little modification wherein methanol was taken in place of water for suspension of TiO_2_. 3 gm of Degussa P25 TiO_2_ has been suspended in 100 ml of methanol, followed by the addition of required amount of Cobalt (II) Nitrate Hexahydrate and urea solution. The obtained slurry was well stirred for 2 h followed by ultrasonication for 10 minutes and kept at rest for 24 hours. The obtained slurry has been thoroughly washed with distilled water for removal of undoped ions before drying in hot air oven at 100 °C for overnight. The solid particles were grounded in agate mortar followed by calcination at 400 °C for 2 hours in muffle furnace. The codoped TiO_2_ photocatalyst were prepared with codopant concentrations of 2 wt% and were denoted by TCo_x_N_y_ (x = 0.5, 1 and 1.5 and y = 1.5, 1.0 and 0.5 respectively), where x and y are the wt% of Co and N, respectively.

To study the crystal structure and crystallinity of Co-N-TiO_2_ nanoparticles, X-ray diffraction (XRD) analysis was performed on X’Pert PRO (D8 Advance) XRD diffractometer using Cu K*α* (*λ* = 0.15406 nm) radiation. To investigate the light absorption and optical band gap of the synthesized TiO_2_ nanoparticles, the UV-vis absorption spectra were obtained with a UV-vis spectrophotometer for determining the binding energy with respect to Co and N. Fourier transform infrared (FTIR) spectra was recorded on Agilent technologies cary 600 series. Transmission Electron Microscope (TEM) characterization was done by using an FEI TECNAI G2 20 - TWIN 120 kV and Scanning Electron Microscope (SEM) characterization was done by using an FEI-Nova nano SEM-450. Energy-dispersive X-ray spectroscopy (EDX) attached to the SEM was used to determine the composition of elements. Surface composition and electronic structures were analyzed by X-ray photoelectron spectroscopy using an ULVAC-PHI (model: PHI5000VersaProbeII) XPS system.

### Photocatalytic degradation of Bisphenol-A (BPA)

The photocatalytic activity of codoped photocatalysts were examined for the degradation BPA under solar light followed by adsorption-desorption equilibrium in dark for 20 mins. During the solar light irradiation experiments, stirring was maintained by magnetic stirrer to manage the solution homogeneous. A fixed amount of sample was withdrawn at different time intervals over 140 min and placed into different syringes and filters. The BPA concentration was determined using a UV-vis spectrophotometer (Perkin Elmer Lambda 35UV-Vis spectrophotometer) at λ_max_ = 277 nm followed by centrifugation for the separation of catalyst. The percentage photodegradation of BPA was calculated using the equation (4):

4 $${\rm{Percentage}}\,{\rm{photodegradation}}\,{\rm{of}}\,{\rm{BPA}}=\frac{{{\rm{C}}}_{{\rm{o}}}-{\rm{C}}}{{{\rm{C}}}_{{\rm{o}}}}\times 100$$

Where, C_o_ is the initial concentration of BPA and C is the concentration of BPA at time ‘t’.

For complete mineralization of the BPA solution, the total organic carbon (TOC) content was estimated using Schimadzu model TOC-V_CPH_ Total Organic Carbon Analyzer.

Gas chromatography- mass spectroscopy (GC-MS) of the photo-degraded BPA wastewater was carried out to identify the various intermediates produced during photocatalytic degradation of BPA in wastewater. The GC-MS analysis was carried out by Perkin Elmer Clarus 500 GC coupled with a Perkin Elmer Clarus 500 mass spectrometer.

## Supplementary information

Supplementary Figures
