# Supplementary material for: Photocatalytic Degradation of Bisphenol-A using N, Co Codoped TiO2 Catalyst under Solar Light
Source: Sci Rep. 2019 Jan 24;9:765. doi: 10.1038/s41598-018-38358-w (PMC6346092; doi:10.1038/s41598-018-38358-w)

Photocatalytic Degradation of Bisphenol-A using N, Co Codoped TiO_2 Catalyst under Solar Light

Alok Garg[1*], Tejasvi Singhania[2], Ashutosh Singh[3], Shilpa Sharma[4], Sonam Rani[2], Ananya Neogy[5], Shri Ram Yadav[5], Vikas Kumar Sangal[6], Neha Garg[3*],

[1]Department of Chemical Engineering, Thapar Institute of Engineering & Technology, Patiala-147004, India
[2]School of Chemistry and Biochemistry, Thapar Institute of Engineering & Technology, Patiala-147004, India
[3]School of Basic Sciences, Indian Institute of Technology Mandi-175005, India
[4]School of Science, Sandip University, Nashik, Maharashtra-422213, India
[5]Department of Biotechnology, Indian Institute of Technology Roorkee-247667, India
[6]Department of Chemical Engineering, Malaviya National Institute of Technology, Jaipur, India

*Corresponding Author

Email: alok.garg.chem@gmail.com, neha@iitmandi.ac.in

Supplementary Figure S1: XPS of pure TiO_2 sample (a) whole, (b) Ti 2p, (c) O 1s, and (d) C 1s

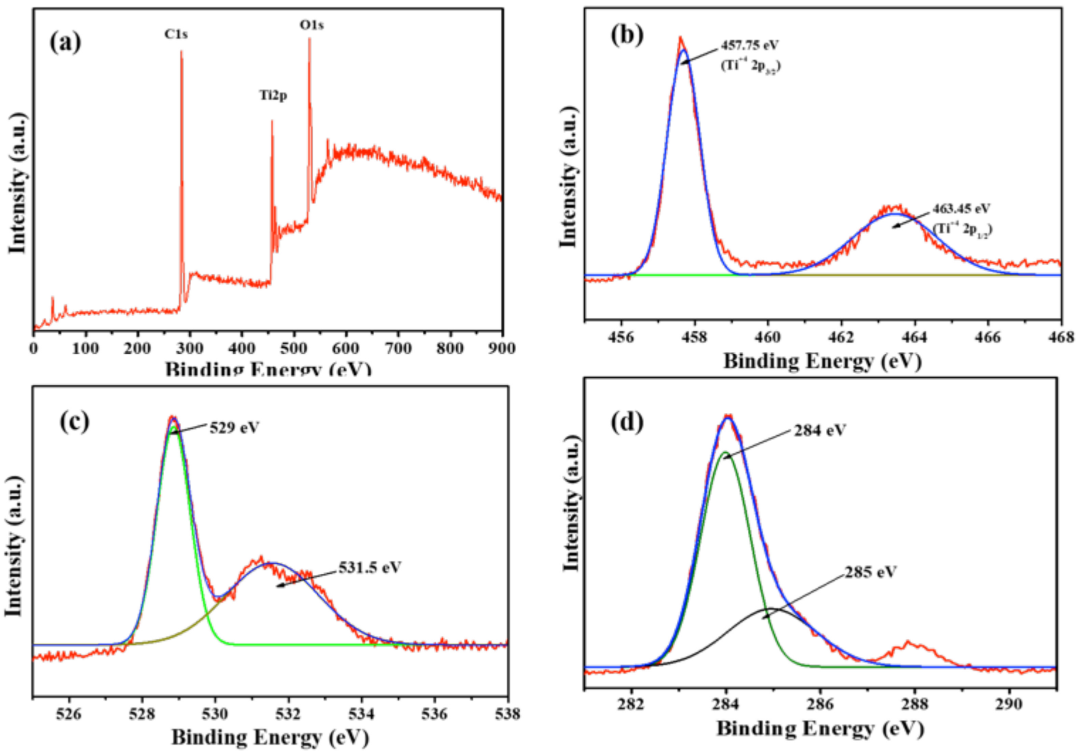

Supplementary Figure S2: XPS of $TCo_{0.5}N_{1.5}$ sample (a) whole, (b) Ti 2p, (c) O 1s, (d) C 1s, (e) Co 2p3 and (f) N 1s

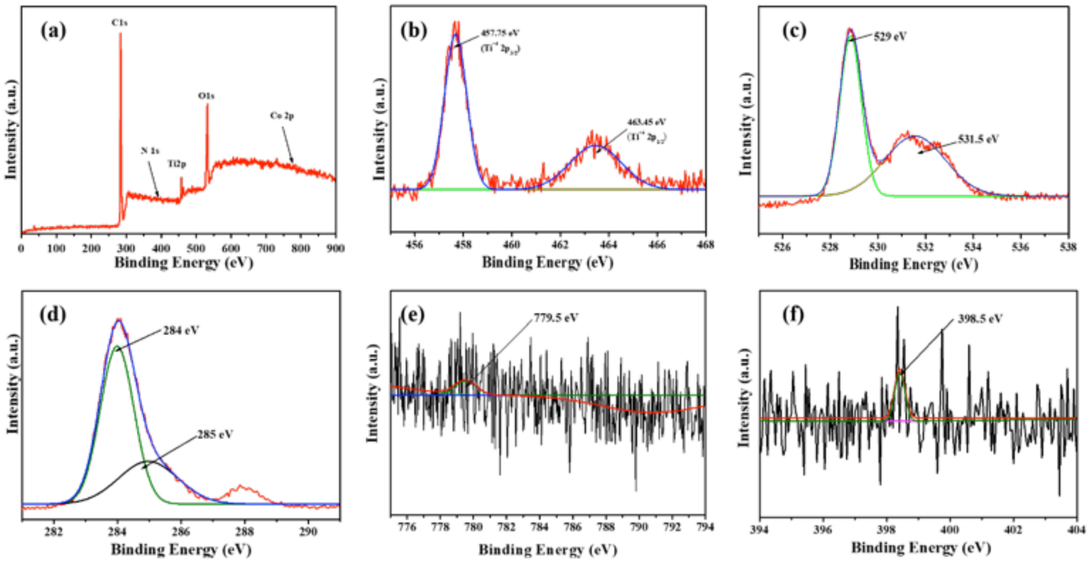

Supplementary Figure S3: XPS of $TCo_{1.5}N_{0.5}$ sample (a) whole, (b) Ti 2p, (c) O 1s, (d) C 1s, (e) Co 2p3 and (f) N 1s

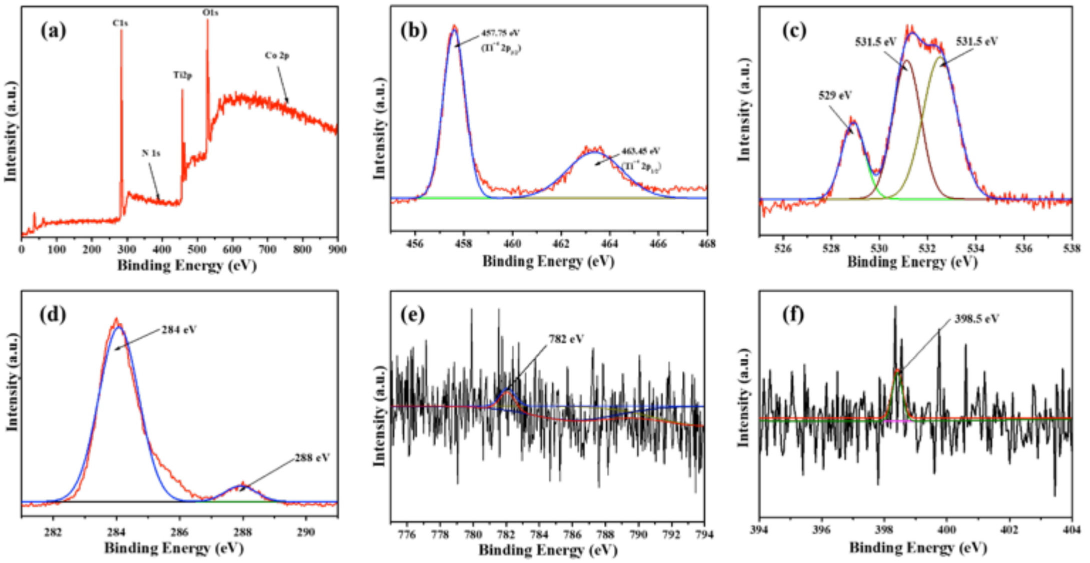

Supplement: Supplementary file 1 — Supplementary Figures [file 41598_2018_38358_MOESM1_ESM.pdf]
